# Ballistic Resistance of Honeycomb Sandwich Panels under In-Plane High-Velocity Impact

**DOI:** 10.1155/2013/892781

**Published:** 2013-09-25

**Authors:** Chang Qi, Shu Yang, Dong Wang, Li-Jun Yang

**Affiliations:** State Key Laboratory of Structural Analysis for Industrial Equipment, School of Automotive Engineering, Dalian University of Technology, B1211 Innovation Park, No. 2 Linggong Road, Ganjingzi District, Dalian 116024, China

## Abstract

The dynamic responses of honeycomb sandwich panels (HSPs) subjected to in-plane projectile impact were studied by means of explicit nonlinear finite element simulations using LS-DYNA. The HSPs consisted of two identical aluminum alloy face-sheets and an aluminum honeycomb core featuring three types of unit cell configurations (regular, rectangular-shaped, and reentrant hexagons). The ballistic resistances of HSPs with the three core configurations were first analyzed. It was found that the HSP with the reentrant auxetic honeycomb core has the best ballistic resistance, due to the negative Poisson's ratio effect of the core. Parametric studies were then carried out to clarify the influences of both macroscopic (face-sheet and core thicknesses, core relative density) and mesoscopic (unit cell angle and size) parameters on the ballistic responses of the auxetic HSPs. Numerical results show that the perforation resistant capabilities of the auxetic HSPs increase as the values of the macroscopic parameters increase. However, the mesoscopic parameters show nonmonotonic effects on the panels' ballistic capacities. The empirical equations for projectile residual velocities were formulated in terms of impact velocity and the structural parameters. It was also found that the blunter projectiles result in higher ballistic limits of the auxetic HSPs.

## 1. Introduction

As a new type of composite structure, lightweight sandwich panels with metallic honeycomb filler have been widely used as weight-efficient components, and are offering a wide range of potential applications in automotive, aerospace and military industries due to their great load-bearing capabilities in association with excellent energy dissipating performance. In certain circumstances, these composite sandwich panels may encounter extreme loading conditions such as projectile impact. For instance, sandwich type vehicle armor could be impacted by bullets or blast debris. In another case, jet engine nacelles may be subjected to impact by birds, hailstones, and runaway debris. Depending on the impact energy, the resulting damage to the honeycomb sandwich panels (HSPs) ranges from face-sheet indentation to complete perforation. One important parameter to describe a structure's ability to withstand projectile impact is its ballistic limit, which is defined as the maximum projectile velocity that a structure can withstand without being perforated [1]. Therefore, the study of ballistic behavior of the HSPs is a critical demand of advanced industries.

Substantial efforts have been devoted to the projectile impact behavior of metallic honeycomb panels through experimental [2–8], analytical [9–12], and numerical methods [7, 13–15]. Goldsmith and coworkers [2–4] conducted a series of experimental tests for the perforation characteristics of aluminum honeycombs and honeycomb sandwich plates impacted by various strikers. Effects of initial velocity, size, and shape of the strikers and the boundary conditions on the ballistic limits and energy dissipation of these structures were investigated. Mines et al. [5] conducted low-velocity impact tests on square panels with polymeric composite skins and honeycomb core. They observed that higher impact velocities tend to increase the energy absorption of the panels, which is attributed to an increase in the core crush stress and skin failure stress at high strain rates. Nia et al. [6] performed experimental tests for evaluation of the ballistic limit velocity, energy dissipation, and damage zone of aluminum honeycombs against cylindrical steel projectiles. They found that folding of the cell walls plays the greatest role in energy dissipation. Meanwhile, an analytical solution for the ballistic limit of a honeycomb plate with metallic face-sheets subjected to normal projectile impact was presented by Hoo Fatt and Park [9], both blunt and spherical projectiles were considered. Later, Lin and Hoo Fatt [10] developed more refined analytical models for the perforation of honeycomb plate with composite face-sheets. Liaghat et al. [11] introduced an analytical model to predict the ballistic limit of metallic honeycombs impacted by cylindrical projectiles. More recently, Feli and Pour [12] presented an analytical model for simulating perforation of composite sandwich panels with honeycomb core subjected to high-velocity impact. The residual velocity, perforation time, velocity-time history of projectile, and absorbed energy of sandwich panel computed by the analytical model have an acceptable consistency with experimental and numerical results. Numerical methods, typically the finite element analysis (FEA), have been employed by some researchers to study in depth the perforation process of HSPs subjected to low-velocity [13, 14] and high-velocity [15] impacts. 

All the above-mentioned studies have been focused on the out-of-plane (axial) ballistic impact behavior of a honeycomb since the honeycomb is more effective in energy absorption under out-of-plane impact than under in-plane impact [16]. However, in some applications, such as using a honeycomb block as vehicle armor against blast debris or as aircraft body panel against bird strike, the honeycomb could be loaded along any direction [17]. As such, the in-plane ballistic response of a honeycomb also needs to be studied along with its out-of-plane behavior. In fact, in-plane impact behavior of honeycombs has drawn increasing interest in the recent years [17–22], although much attention has been paid to the dynamic response of the honeycombs under distributed loads exerted by, for example, a rigid wall. Compressive stress or crushing strength of the honeycombs is the main focus in all these studies, since it directly determines the energy absorption capabilities of such structures. For instance, Hu and Yu [17] derived an analytical formula for the dynamic in-plane crushing strength of regular hexagonal honeycombs in terms of impact velocity and cell walls' thickness ratio. Qiu et al. [22] found that the average stress increases with the impact velocity, the density of the base material, and the relative density of the lattice. Liu and Zhang [19] demonstrated the plateau stress for triangular or quadratic honeycombs also obeys the Reid-Peng equation, increasing with the impact velocity by a square law. Zou et al. [23] showed that the one-dimensional shock theory [24, 25], which was based on an equivalent rigid-perfectly plastic-locking stress-strain curve, tends to overestimate slightly the crushing stress and energy absorption. In addition, several researchers found that cell shape [19, 22] and arrangement patterns [19] have a great influence on the deformation modes and energy absorption of the honeycombs under in-plane dynamic crushing. More recently, Ajdari et al. [18] showed that decreasing the relative density in the direction of crushing enhances the energy absorption of honeycombs at early stages of crushing. In contrast to these in-depth studies of honeycombs under in-plane distributed loads, whereas limited attention has been paid on the dynamic response of honeycombs or honeycomb sandwiches subjected to in-plane concentrated loads, especially high-velocity loads such as in the ballistic impact scenarios.

The present study aims to assess the ballistic resistant performance of the honeycomb sandwich panels (HSPs) against high-velocity in-plane projectile impact using numerical methods. Towards this end, finite element (FE) models were established in the work reported herein to provide information about the impact behavior, for example, failure modes of the HSPs and the velocity evolution of the projectile during the impact process. Ballistic capacity indices including the residual velocity, ballistic limit, and contact time, as well as energy absorption were obtained from FE simulations. 

In many honeycomb material papers, cell configuration is considered but the loads are mostly distributed. Therefore, in this paper, particular attention has been given to the influence of cell configuration of the honeycomb core, which is determined by the cell angle  *λ*, on the ballistic resistance of the sandwich panels. Furthermore, parametric studies are performed to evaluate the effects of several parameters, for example, impact velocity, honeycomb relative density, core and face-sheets thicknesses, and cell angle and size on the ballistic behavior of the HSPs. The primary outcome of this study is design information for such panels to be employed as armors against ballistic impact loads.

## 2. Problem Description

**Figure 1:**
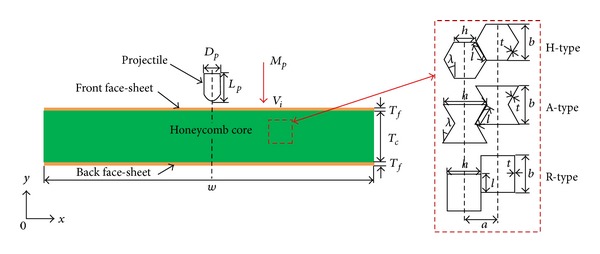
Diagrammatic sketch of the honeycomb sandwich panels (HSPs) under in-plane projectile impact.

The diagrammatic sketch of the impact problem considered in this study is shown in Figure 1. The baseline target sandwich structure consists of a honeycomb core of thickness *T*_*c*_ = 50 mm sandwiched between two identical thin face-sheets with thickness *T*_*f*_ = 1.0 mm. The sandwich panel is impacted by a lightweight hemispherical nose projectile with diameter *D*_*p*_ = 15 mm and length *L*_*p*_ = 25.5 mm. The projectile has a mass of *M*_*p*_ = 1.44 g and an initial impact velocity *V*_*i*_, which varies from 150 m/s to 350 m/s. The width of the sandwich panel has been deliberately set long (*w* = 300 mm) to eliminate any influence of boundary conditions, even though such influence is usually negligible in high-velocity impacts [15]. The unit cell of the honeycomb core is also depicted in Figure 1. The parameters defining the hexagon geometry include: the horizontal wall length *h*, inclined wall length *l*, cell angle *λ*, wall thickness *t*, and cell depth *d* = 2 mm, which is perpendicular to the *X*-*Y* plane. Variation of the cell angle *λ* results in three cell configurations of regular (H-type) (*λ* = 30°), rectangular-shaped (R-type) (*λ* = 0°), and reentrant or auxetic [26] (A-type) hexagons (*λ* = −30°). For comparison purpose, the cell numbers along the horizontal (*N*_*x*_ = 50) and vertical (*N*_*y*_ = 7) directions comprising the honeycomb core are the same for all three cell configurations. In the current circumstance, the size of a repetitive baseline unit of the honeycomb is *a* × *b* = 6 mm × 6.928 mm (Figure 1), corresponding to an H-type unit cell with *l* = *h* = 4 mm, an R-type unit cell with *l* = 3.464 mm, *h* = 6 mm and an A-type unit cell with *l* = 4 mm, *h* = 8 mm, respectively.

## 3. Computational Models

### 3.1. Finite Element Modeling

In this part of the study, we have developed finite element (FE) models of the HSPs with the three hexagonal cell shapes to simulate their dynamic behavior under in-plane ballistic impact. The CAD models and FE models of the HSPs are generated in ANSYS [27] using APDL (ANSYS Parametric Design Language). The explicit nonlinear program LS-DYNA 970 [28] is adopted as FE solver, and the postprocessor LS-PREPOST is employed for visualization and data processing.

**Figure 2:**
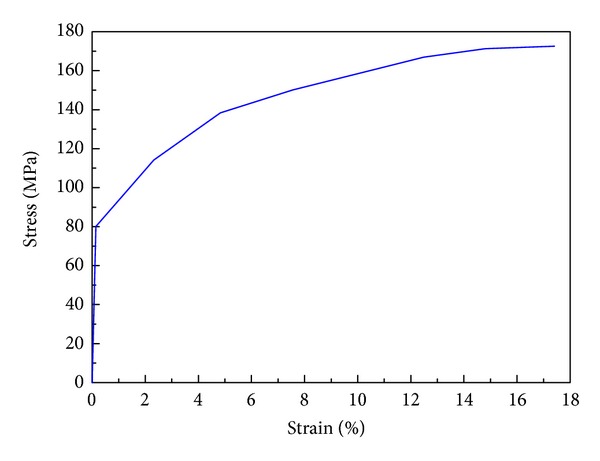
Tensile stress-strain curve of HSP material AA6060 T4.

The face-sheet material and the matrix material of the honeycomb core are aluminum alloys AA6060 T4 with Young's modulus *E* = 68.2 GPa, initial yield stress *σ*_*y*_ = 80 MPa, ultimate stress *σ*_*u*_ = 173 MPa, Poisson's ratio *ν* = 0.3, and mass density *ρ* = 2700 kg/m^3^. The tensile stress-strain curve is shown in Figure 2. In the FEA, the material behavior is based upon the piecewise linear plasticity material model, MAT 24, provided by LS-DYNA. The strain rate effect is neglected in the FE modeling as the aluminum alloy is strain rate insensitive [29]. The projectile is modeled by MAT 20 (MAT_RIGID) in LS-DYNA assuming no damage during the impact process.

The face-sheets and honeycomb wall are modeled with Belytschko-Tsay 4-node shell element with five integration points through the thickness. Element size of 0.5 mm for the damage region nearest the projectile trajectory and 1.0 mm for the rest part of the structure have been proven to be sufficient to produce reliable results through a mesh sensitivity analysis. Offsets are defined between the face-sheets and the honeycomb core to eliminate initial penetration of the shell elements. A critical plastic failure strain of 0.8 is defined for all elements, above which the shell element is eroded and excluded from the calculation. It is also considered, in the FE models, that some cell walls have double thickness due to the manufacturing process [17]. The projectile is modeled by the 8-node constant stress solid element. All the nodes in each FE model are constrained from the out-of-plane degrees of freedom to ensure the plane strain state of deformation. The initial velocity of the projectile is exerted by the INITIAL_VELOCITY_RIGID_BODY card in LS-DYNA.

**Figure 3:**
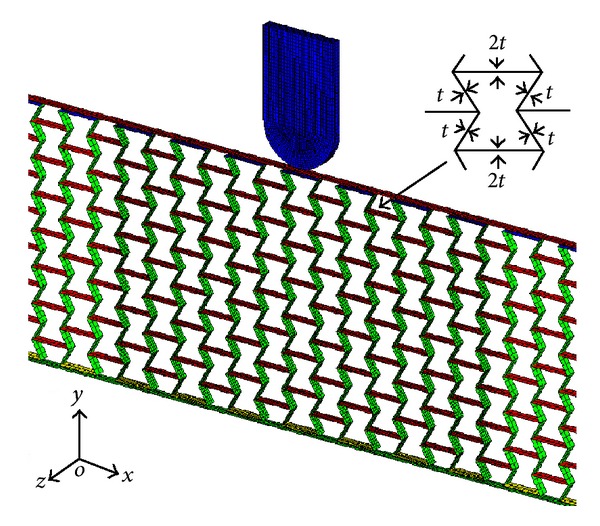
The representative FE model of the HSP (part) under projectile impact.

Contact modeling is critical for predicting the ballistic response of armor structures [14, 30]. In this study, an automatic single-surface contact is defined for the honeycomb core to account for the extensive contacts between deformable honeycomb cell walls during the projectile impact process. An automatic surface-to-surface contact is set between the projectile and the HSP. Moreover, the contact algorithm CONTACT_TIED_SURFACE_TO_SURFACE_OFFSET in LS-DYNA is selected for modeling the adhesive bonding between the honeycomb core and the face-sheets of the sandwich panel [31]. For all possible contact interfaces, a friction coefficient of 0.1 is adopted in both static and dynamic friction conditions. A part of representative FE model of the HSP subjected to projectile impact is shown in Figure 3.

### 3.2. Relative Density of Honeycomb Core

The most common honeycombs are manufactured by the expansion technique from the equidistantly-glued metal foil [17]. Therefore, the horizontal cell walls have the double thickness (2*t*) of the inclined walls (*t*) (Figure 3). The relative density of honeycomb filled by hexagon cells can be derived as

(1) $$\begin{array}{c} \overset{-}{\rho }=\frac{\rho^{*}}{\rho_{s}}=\frac{t\left(h/l+1\right)/l}{\cos\lambda \left(h/l+\sin\lambda \right)}, \end{array}$$

where ρ* and *ρ*_*s*_ are the densities of hexagon honeycomb and the matrix material, respectively. Based on (1), the relative densities of honeycombs filled by unit cells with the three types of configurations, that is, H-type (*λ* = 30°), R-type (*λ* = 0°), and A-type (*λ* = −30°) hexagons can be achieved, respectively, as

(2) $$\begin{array}{c} {\overset{-}{\rho }}_{H}=\frac{\rho_{H}^{*}}{\rho_{s}}=\frac{4}{\sqrt{3}}\frac{t}{l}\left(\frac{h+l}{2h+l}\right),\\ {\overset{-}{\rho }}_{R}=\frac{\rho_{R}^{*}}{\rho_{s}}=\frac{t}{h}\left(1+\frac{h}{l}\right),\\ {\overset{-}{\rho }}_{A}=\frac{\rho_{A}^{*}}{\rho_{s}}=\frac{4}{\sqrt{3}}\frac{t}{l}\left(\frac{h+l}{2h-l}\right). \end{array}$$

For comparison purpose, the horizontal wall length *h* and inclined wall length *l* are fixed herein for each type of cell configuration as specified in Section 2. Variation of relative density of the honeycomb core is realized by adjusting the cell wall thickness. For the baseline designs, the core relative density is chosen as $\overset{-}{\rho }=0.15$, the corresponding cell wall thicknesses for the three cell configurations are then determined to be *t*_*H*_ = 0.39 mm, *t*_*R*_ = 0.33 mm, and *t*_*A*_ = 0.26 mm, respectively, using (2).

## 4. Ballistic Resistance Comparison of HSPs with Different Cell Configurations

In this study, it is of special interest to investigate the influence of unit cell configuration of the honeycomb core, that is, H-type, R-type, or A-type hexagons, on the perforation resistant capability of the entire sandwich panel. To this end, numerical simulations were carried out for the three types of HSPs subjected to various projectile impact velocities from 150 m/s to 350 m/s. Residual velocity of the projectile was recorded in each simulation. Using these data, two key quantitative indices, namely, the ballistic limit *V*_*b*_ and the perforation energy *E*_*p*_, were analyzed in detail for each type of HSP to evaluate its ballistic resistant performance and energy dissipating capacity. The ballistic limit of a sandwich structure can be defined as the velocity when the projectile is either stuck in the back face-sheet or exits with a negligible velocity, while the perforation energy is essentially the energy absorption by the panel during perforation.

### 4.1. Comparison of Ballistic Limit and Energy Absorption

**Figure 4:**
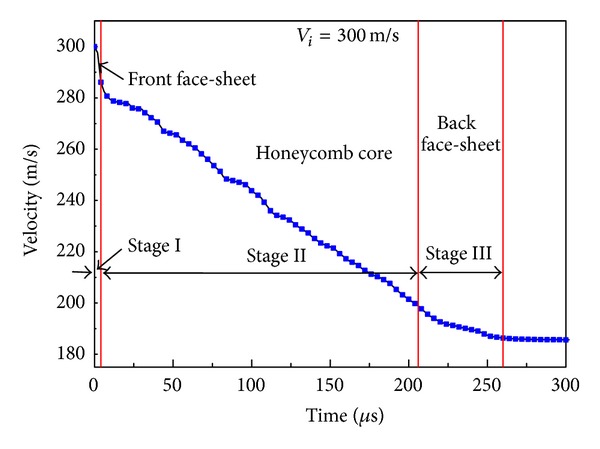
The evolution of projectile velocity in perforating the A-type HSP at *V*_*i*_ = 300 m/s.

As representative of each impact scenario, the evolution of the projectile velocity during the perforation of the A-type HSP at an impact velocity *V*_*i*_ = 300 m/s is depicted in Figure 4. A total perforation time is estimated to be 260 *μ*s, which can be defined as the time between the contact of the projectile with the front face-sheet and the instant at which the projectile completely penetrates the sandwich panel. Three different stages in the perforation process can be identified corresponding to the three components of the sandwich panel, that is, front face-sheet, honeycomb core, and back face-sheet. In stage I (0–4 *μ*s), the front face caused a sudden drop in velocity at the beginning of the impact event, so that the projectile reached the honeycomb core at a velocity of nearly 287 m/s. In stage II (4–206 *μ*s), the velocity continuously decreased as the projectile went through the core, when the projectile reached the back face-sheet, its velocity was about 199 m/s. In stage III (206–260 *μ*s), another drop in velocity was caused by the back face with a residual velocity of nearly 186 m/s. The projectile lost 62% of its initial kinetic energy during the impact process. The front and back face-sheets absorbed 12% and 9% of the total absorbed energy, respectively, and the honeycomb core absorbed 79%. This analysis was performed for each numerical simulation case and the results are discussed in the following.

**Figure 5:**
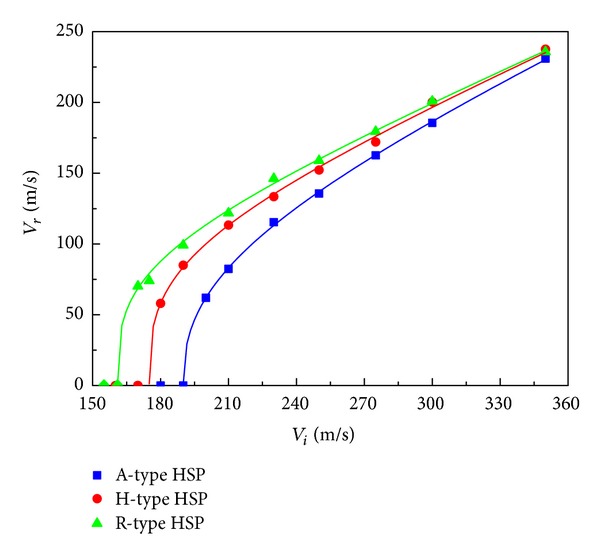
Residual velocity versus impact velocity of projectile for three types of HSPs.

Variations of residual velocity of the projectile as a function of the impact velocity for the three HSPs are presented in Figure 5. The fitting curves shown in Figure 5 were calculated using the Lambert-Jonas model [32] which relates residual velocity *V*_*r*_, to impact velocity *V*_*i*_, by means of the following equation:

(3) $$\begin{array}{c} V_{r}=A\cdot {\left({V_{i}}^{p}-{V_{b}}^{p}\right)}^{1/p}, \end{array}$$

where *V*_*b*_ is the ballistic limit; *A*  and *p* are empirical parameters. In this study, the values of *A*, *p* and *V*_*b*_ are estimated by the least square fitting method. 

Table 1 provides the values of *A* and *p* and the ballistic limits estimated by (3) for the three HSPs. The A-type HSP yields the highest ballistic limit, which is estimated to be 190 m/s according to (3). The R-type HSP results in a ballistic limit value of 161 m/s, and the H-type HSP's performance is in between with a ballistic limit of 175 m/s, under the in-plane impact of the projectile considered. It is also observed from Figure 5 that the three ballistic curves tend to converge with increasing impact velocity. For instance, at an impact velocity of 210 m/s, the residual velocities for the A-type, H-type, and R-type HSPs are 82.4 m/s, 113.4 m/s, and 121.8 m/s, respectively. When the impact velocity is increased to 350 m/s, the residual velocities are 231.0 m/s, 237.6 m/s, and 235.8 m/s, respectively. This implies that the influence of cell configuration of the honeycomb core on the perforation resistance of the HSP becomes smaller with increased impact velocity of the projectile. 

**Figure 6:**
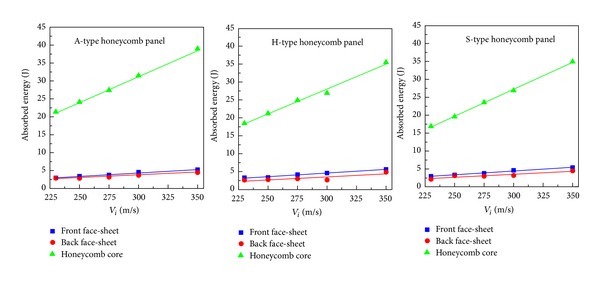
Absorbed energy versus impact velocity for the three types of HSPs.

Figure 6 provides, further, the energy absorbed by the three components of each sandwich panel at different impact velocities. For the range of velocity examined, the energy absorption of each component increases almost linearly along with the increase of impact velocity. Different from the case of out-of-plane ballistic impact on honeycomb sandwiches when the face-sheets play a leading role in energy absorption [15], it was found in this study that the honeycomb core absorbs the vast majority of the perforation energy when the HSPs are subjected to in-plane impacts, as depicted in Figure 6. Moreover, for each type of HSP, the energy absorption of the honeycomb core has a rapid increase pace with the impact velocity more rapidly than that of the face-sheets. In most cases, the front face-sheet absorbs slightly more energy than the back face-sheet. Same findings have been reported for HSPs under out-of-plane projectile impacts [12, 15]. More importantly, the A-type honeycomb core absorbs more energy than the other two types of cores throughout the impact velocity range investigated, although the energy absorption levels of the face-sheets are almost the same for all three HSPs.

### 4.2. Ballistic Impact Process Analysis

To find the reasons behind the ballistic characteristics of the HSPs shown above, in this section, detailed impact processes of the three sandwiches are analyzed at three representative impact velocities of 170 m/s, 190 m/s, and 350 m/s, respectively.

**Figure 7:**
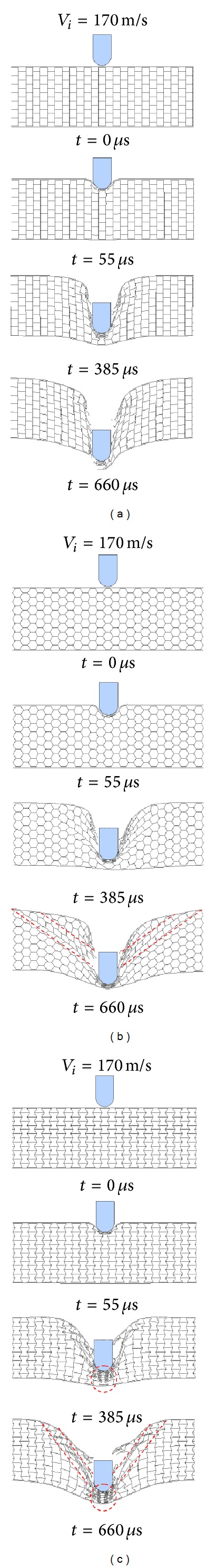
Impact processes of (a) R-type, (b) H-type, and (c) A-type HSPs by the projectile at *V*_*i*_ = 170 m/s.

**Figure 8:**
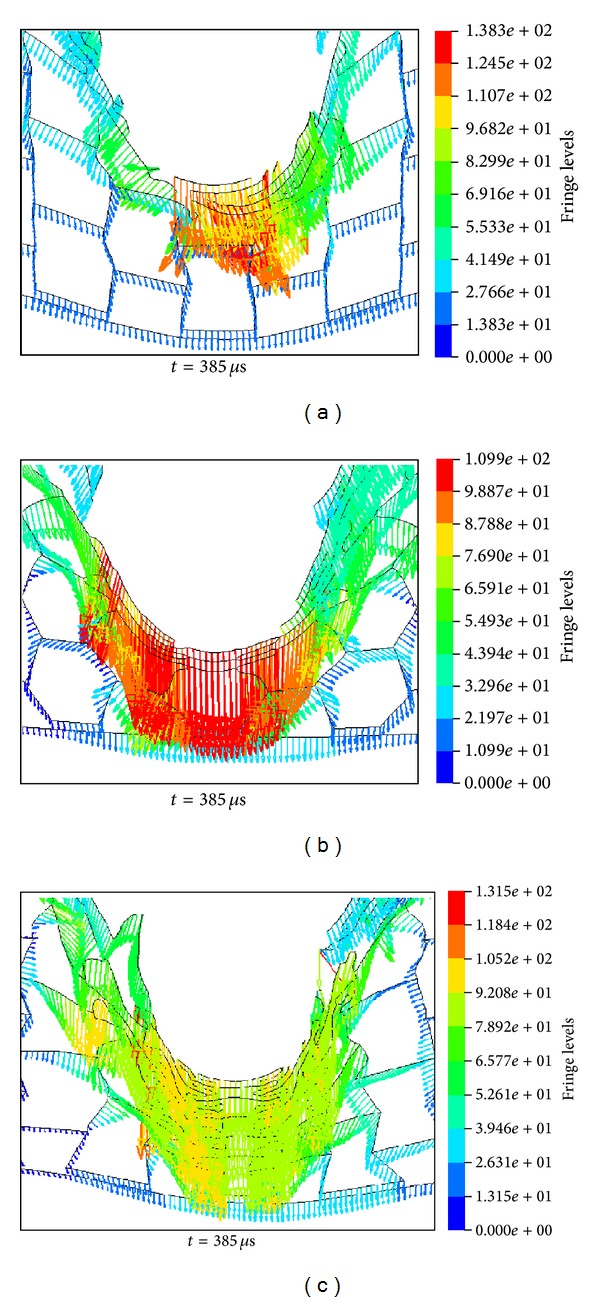
Local instant node velocity (amplitude and direction are represented by length and direction of the arrow, resp.) distribution of (a) R-type, (b) H-type, and (c) A-type honeycomb cores under the projectile impact at *V*_*i*_ = 170 m/s (projectile masked for better view).

**Figure 9:**
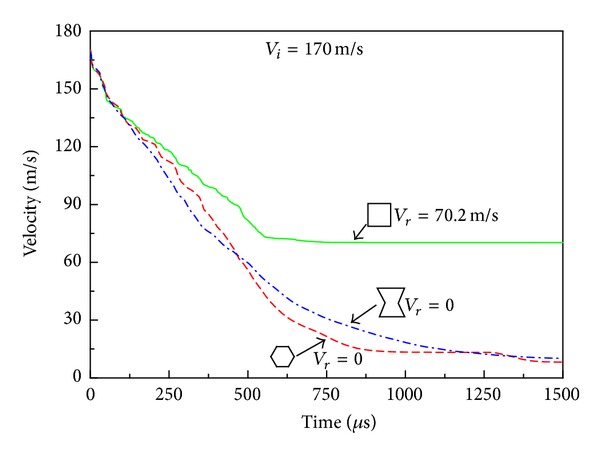
Time history of the transient velocity of the projectile with *V*_*i*_ = 170 m/s. Here *V*_*r*_ is the residual velocity.

Snap-shots of some typical deformation stages of the HSPs under impact velocity *V*_*i*_ = 170 m/s are depicted in Figure 7. At such an impact velocity higher than the ballistic limit of the R-type HSP (*V*_*b*_ = 161 m/s) and lower than that of the H-type (*V*_*b*_ = 175 m/s) and A-type (*V*_*b*_ = 190 m/s) HSPs, the R-type panel is fully perforated (Figure 7(a)) with a total perforation time *t*_*p*_ about 700 *μ*s and a residual velocity *V*_*r*_ = 70.2 m/s (Figure 9), while both the H-type and A-type panels are only partially perforated (Figures 7(b) and 7(c)). At the beginning of the impact (e.g., *t* = 55 *μ*s), the responses of the three HSPs are identical with only local indentations around the projectile head while the rest part of the structure remains intact. As the projectile penetrates the front face and starts to interact with the honeycomb core, for example, *t* = 385 *μ*s, due to the difference in the unit cell configuration, the local deformation evolution displays distinct properties, and the dynamic response of the honeycomb cores shows different characteristics. For the R-type honeycomb core with rectangular-shaped hexagon cell, the vertical edges of the unit cells impacted by the projectile are compressed and buckled layer by layer. The cells adjacent to the projectile path endure mainly shear loadings (Figures 7(a) and 8(a)), and the number of unit cells, that is, area of the core material, affected by the projectile is relatively small. In contrast, in the H-type and A-type honeycomb cores, the inclined walls of the unit cells in the projectile path experience primarily tension loads (Figures 7(b), 7(c), 8(b), and 8(c)). These tension loads are transmitted through connected cells to the remote area of the core away from the projectile path, forming the deformation bands marked by dashed lines in Figures 7(b) and 7(c). This type of load transformation involved more materials and therefore results are higher in energy absorption and ballistic limits. Furthermore, Figure 8(b) clearly shows that when a regular honeycomb is subjected to compressive loading from the projectile, the material compensates by spreading in the directions perpendicular to and away from the direction of the impact. Nevertheless, when the reentrant cell honeycomb is compressed, the specimen displays dynamic inhomogeneity and negative Poisson's ratio characteristics. As seen from Figure 8(c), the reentrant cells contract laterally and the material flows into (compresses towards) the vicinity of the impact. This creates an area of denser material directly below the point of impact (marked by dashed circles in Figure 7(c)) and results in additional resistance to the ballistic impact. This so-called “indentation resistance enhancement” of auxetic material has already been found in static indentation testing such as the traditional hardness test as shown in [33], and it has not yet been reported in dynamic impact scenarios as in the current circumstance.

**Figure 10:**
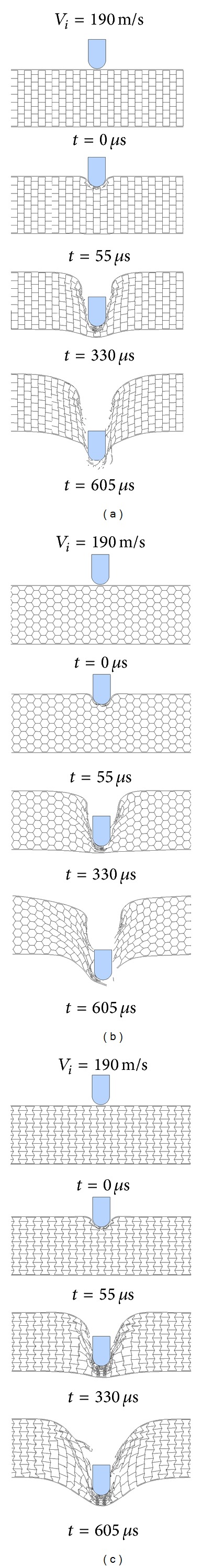
Impact processes of (a) R-type, (b) H-type, and (c) A-type HSPs by the projectile at *V*_*i*_ = 190 m/s.

**Figure 11:**
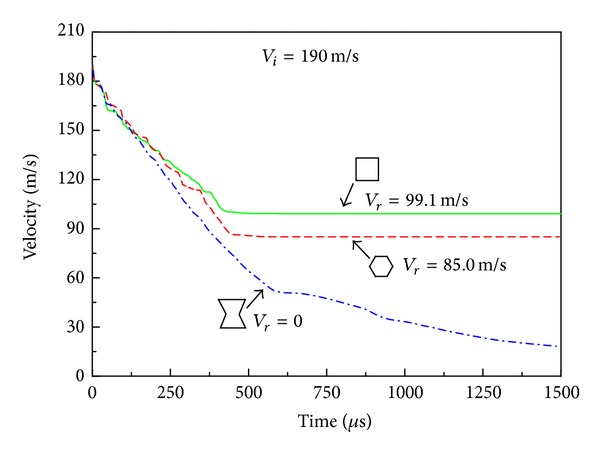
Time history of the transient velocity of the projectile with *V*_*i*_ = 190 m/s. Here *V*_*r*_ is the residual velocity.

When the impact velocity is increased to *V*_*i*_ = 190 m/s, both the R-type and H-type HSPs are completely penetrated (Figures 10(a) and 10(b)) with a residual velocity of 99.1 m/s and 85.0 m/s, respectively (Figure 11). The A-type HSP, however, can still withstand the projectile without being perforated due to the material concentration around the tip of the projectile resulting from the auxetic property of the reentrant honeycomb core (Figure 10(c)). As a result, the projectile velocity decreases more and faster when it penetrates through the auxetic HSP than the other two types of HSPs under the same initial velocity (Figure 11). 

**Figure 12:**
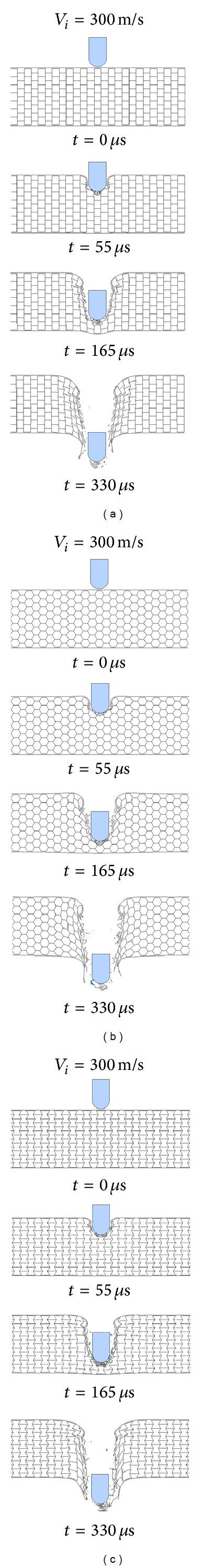
Perforation processes of (a) R-type, (b) H-type, and (c) A-type HSPs by the projectile at *V*_*i*_ = 300 m/s.

**Figure 13:**
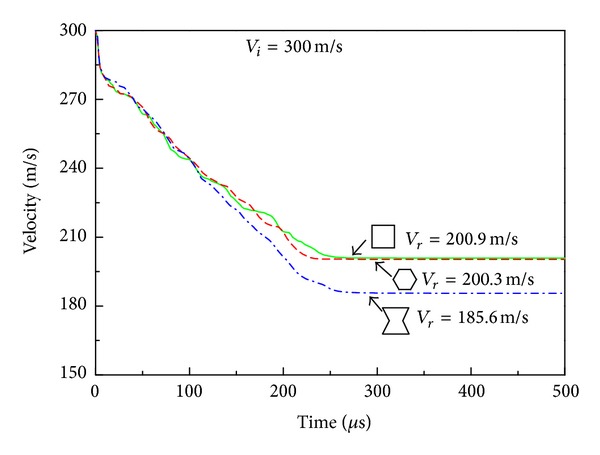
Time history of the transient velocity of the projectile with *V*_*i*_ = 300 m/s. Here *V*_*r*_ is the residual velocity.

Finally, if the impact velocity is further increased to *V*_*i*_ = 300 m/s, all three HSPs are completely perforated (Figure 12). At such a high impact velocity, local damage is the predominant failure mode of the honeycomb core. Neighboring cells to the ones along the projectile path do not have adequate time to react before the projectile passes through the panel. Therefore, the cell configuration of the honeycomb core has little effect on the ballistic characteristic of the sandwich panel. As a result, the residual velocity of the projectile are almost the same for the R-type (*V*_*r*_ = 200.9 m/s) and H-type (*V*_*r*_ = 200.3 m/s) HSPs, whereas the A-type sandwich still yields a smaller residual velocity of *V*_*r*_ = 185.6 m/s because of a relatively longer perforation time (Figure 13).

## 5. Parametric Study of Auxetic Honeycomb Sandwich Panels (HSPs) with Reentrant Unit Cells

According to our simulation results, the auxetic (A-type) HSP with reentrant unit cells shows the best performance in ballistic resistance among all three HSPs within the range of impact velocity considered, especially in relatively low velocity impact cases. In the subsequent sections, the ballistic characteristics of the auxetic HSP are further studied regarding both macroscopic and mesoscopic parameters of the structure itself as well as the profile of the projectile impacting on it. These parameters include the face-sheet thickness, honeycomb core thickness and relative density, reentrant unit cell angle, cell size and wall thickness, and the nose shape of the projectile. The purpose is to provide detailed information for the ballistic design of such an innovative armor structure.

### 5.1. Effect of Macroscopic Parameters on the Auxetic HSP Ballistic Response

#### 5.1.1. Effect of Face-Sheet and Core Thickness

**Figure 14:**
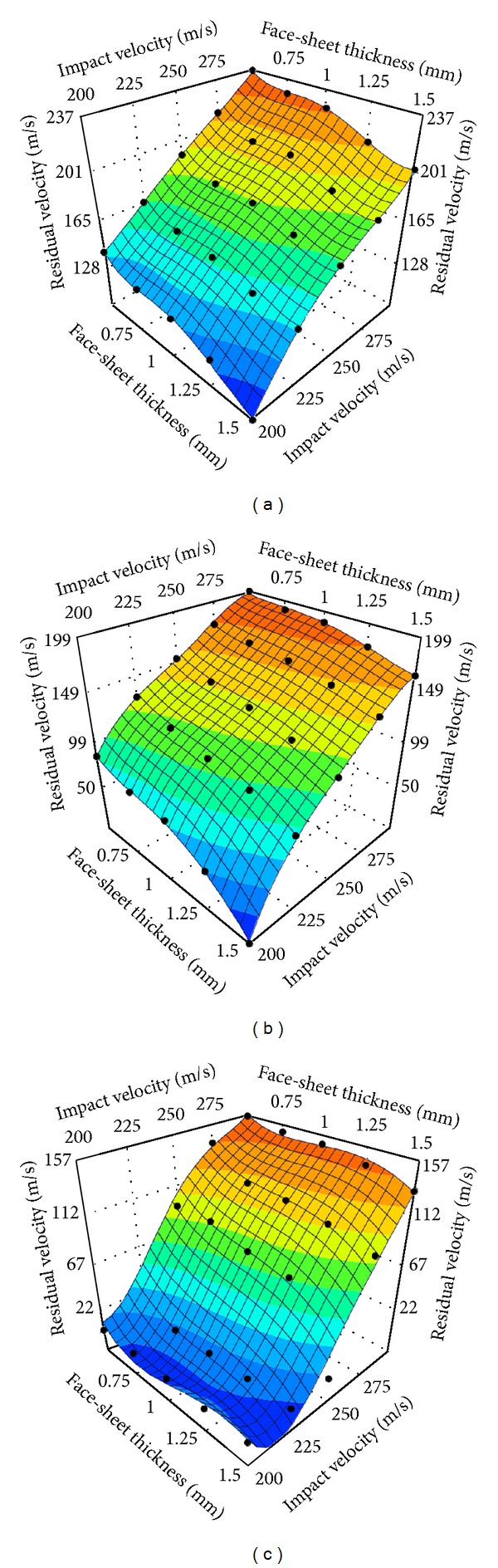
Quartic polynomial approximations of the residual velocity of projectile impact on auxetic HSPs with different face-sheet thicknesses. The bold black dots in the plots show the training points. (a) *T*_*c*_ = 30 mm, (b) *T*_*c*_ = 50 mm, and (c) *T*_*c*_ = 70 mm ($\overset{-}{\rho }=0.15$).

Initially the effects of face-sheet and honeycomb core thicknesses were examined. For this purpose, three groups of numerical tests were conducted. In each group, the auxetic HSPs had identical core thickness (*T*_*c*_ = 30 mm, 50 mm, or 70 mm) but different face thicknesses (*T*_*f*_ = 0.5 mm, 0.75 mm, 1.0 mm, 1.25 mm, and 1.5 mm, resp.) and were impacted by the projectile with five initial velocities of 200 m/s, 225 m/s, 250 m/s, 275 m/s, and 300 m/s, respectively. Residual velocities of the projectile with respect to face thickness and impact velocity, along with the forth order polynomial approximation of the responses are plotted in Figure 14, for each group. As expected, the numerical results show that the residual velocity decreases monotonically with increased face and core thicknesses. The effect of face thickness is more significant for panels with a thinner honeycomb core (Figure 14(a)) than with a thicker core (Figure 14(c)). Moreover, the plots show that the quartic polynomials can approximate the responses appropriately, implying the high nonlinearities of the impact problems. The polynomial functions for projectile residual velocity prediction for the auxetic HSPs with three core thicknesses are provided in the Appendix.

**Figure 15:**
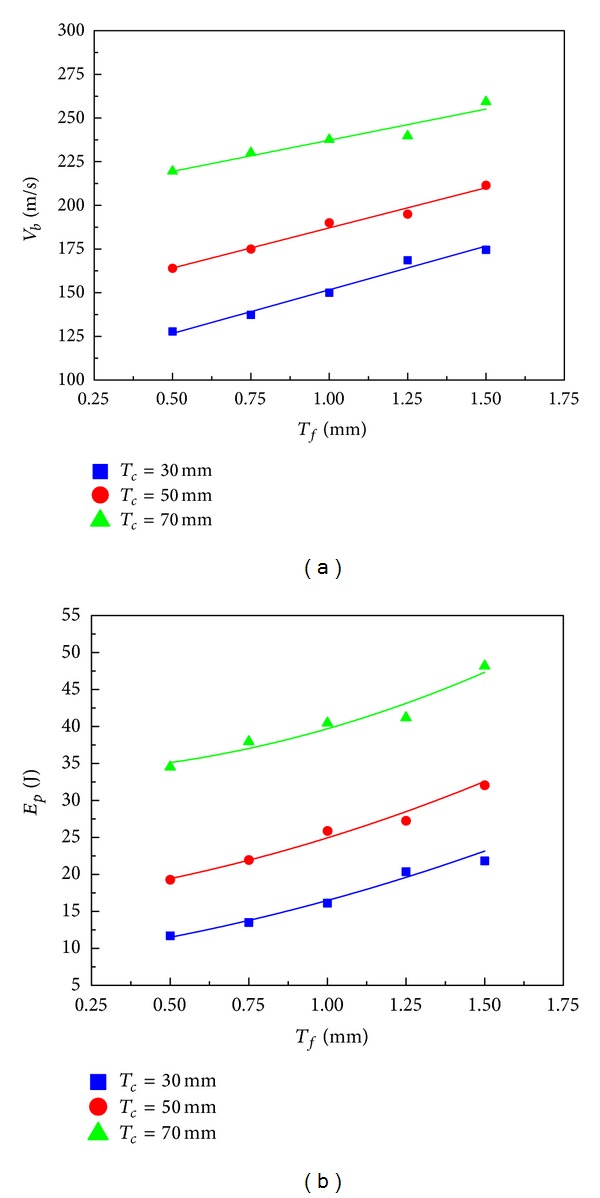
Effects of face-sheet thickness on (a) ballistic limit and (b) minimum perforation energy of the auxetic HSPs with three different core thicknesses ($\overset{-}{\rho }=0.15$).

Based on the impact and residual velocities simulated, the ballistic limit of each panel configuration was obtained using (3) along with the minimum perforation energy. The panels' specifications and results are list in Table 2. The relationship of ballistic limit and face-sheet thickness is shown graphically in Figure 15(a). As we expect, the panels with a thicker core result in higher ballistic limit. For the three core thicknesses considered, the ballistic limit is almost linearly proportional to the face thickness. Using the ballistic limits of the panels with 0.5 mm faces as the baseline, for *T*_*c*_ = 30 mm, the ballistic limit of the panels with 0.75 mm, 1.0 mm, 1.25 mm, and 1.5 mm faces increases by 7.4%, 17.4%, 31.9%, and 36.6%; for *T*_*c*_ = 50 mm, the increases are 6.7%, 15.9%, 18.9%, and 28.9%; while for *T*_*c*_ = 70 mm, the increases are 4.8%, 8.3%, 9.2%, and 18.1%, respectively. This suggests that for HSPs with thinner cores, increasing the face-sheet thickness is an effective way to improve the ballistic limit of the sandwich structure. Moreover, Figure 15(b) indicates that the minimum perforation energy increases with the face-sheet thickness by a square law for all three core thicknesses.

#### 5.1.2. Effect of Core Thickness and Relative Density

**Figure 16:**
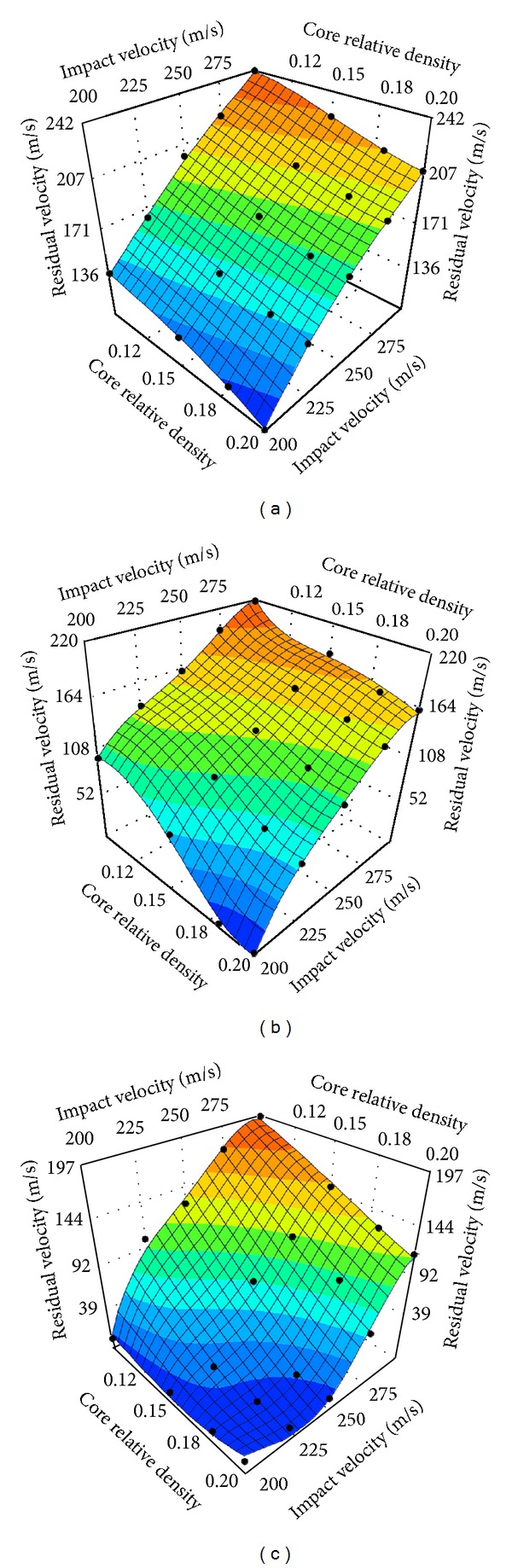
Quartic polynomial approximations of the residual velocity of projectile impact on auxetic HSPs with different core relative densities. The bold black dots in the plots show the training points. (a) *T*_*c*_ = 30 mm, (b) *T*_*c*_ = 50 mm, and (c) *T*_*c*_ = 70 mm (*T*_*f*_ = 1.0 mm).

In order to estimate the effects of honeycomb core thickness and relative density on the ballistic capacity of the panel, evolution of the projectile's residual velocity over a range of initial impact velocities have been presented as functions of core relative density ($\overset{-}{\rho }=0.1$, 0.15, 0.18, and 0.2), for three core thicknesses of 30 mm, 50 mm, and 70 mm, as shown in Figures 16(a)–16(c), respectively. Variation of the core relative density was realized by tuning the unit cell wall thickness. It can be seen from the graphs that the residual velocity is inversely proportional to the core relative density for all three core thicknesses considered, that is, the denser the core, the better the performance. Interestingly, the residual velocity seems to show more nonlinearity in response to the variations of core relative density and impact velocity as the core thickness increases.

**Figure 17:**
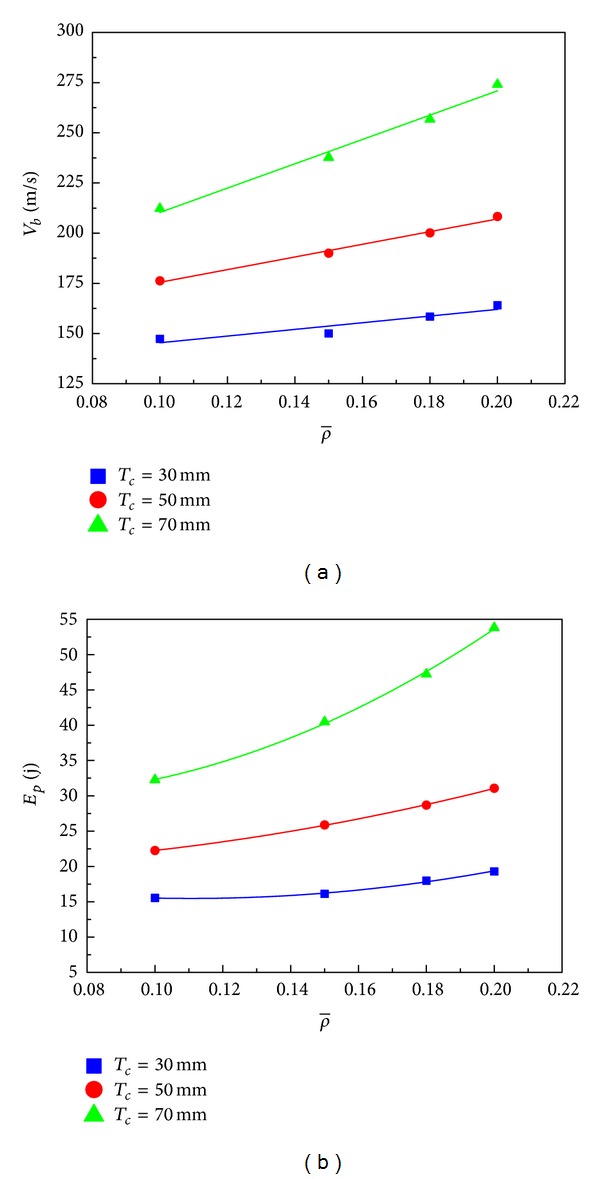
Effects of core relative density on (a) ballistic limit and (b) minimum perforation energy of the auxetic HSPs with three different core thicknesses (*T*_*f*_ = 1.0 mm).

The auxetic HSPs' specifications and the calculated results of ballistic limits and minimum perforation energy values in this section are listed in Table 3.  Figure 17 graphically shows a significant influence of the core relative density on the ballistic limit of the auxetic HSP and reveals the approximate linear relationship between them. We can also observe that the ballistic limit of the sandwich panel with a thicker core increases more rapidly with increased relative density than its counterpart with a thinner core. For instance, with a core thickness of *T*_*c*_ = 30 mm, ballistic limit of the panel increases 11.41% from 147.29 m/s to 164.09 m/s in response to a relative density increase from 0.1 to 0.2. In comparison, ballistic limit of the panel with *T*_*c*_ = 70 mm is increased by up to 29.13% from 212.22 m/s to 274.04 m/s with the same amount of relative density incrimination. From another point of view, the ballistic limits of the three groups are approaching when the density of core is decreasing, which implies that core thickness change has less impact on the ballistic limits of the auxetic HSPs with low density cores. Again, Figure 17(b) clearly illustrates the quadratic relationship between the minimum perforation energy and the core relative density.

### 5.2. Effect of Mesoscopic Parameters on the Auxetic HSP Ballistic Response

#### 5.2.1. Effect of Unit Cell Angle and Wall Thickness

**Figure 18:**
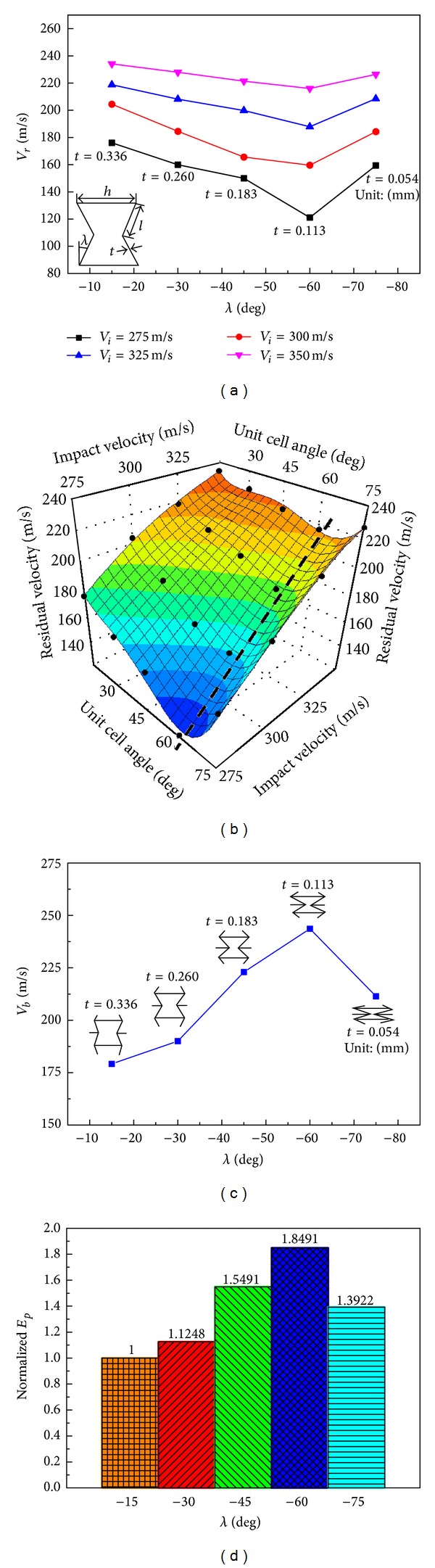
Effects of reentrant unit cell angle and wall thickness on the ballistic responses of auxetic HSPs: (a) residual velocity variations with varied cell angle and wall thickness at different impact velocities; (b) quartic polynomial approximation surface of residual velocity (The bold black dots in the plot show the training points); (c) ballistic limit versus unit cell angle; and (d) normalized minimum perforation energy versus unit cell angle. (All numerical specimens have *T*_*f*_ = 1.0 mm, *T*_*c*_ = 50 mm, $\overset{-}{\rho }=0.15$, *h* = 8 mm, and *l* = 4 mm).

In this section, the influences of unit cell angle *λ* and wall thickness *t* on the ballistic response of the auxetic HSP are discussed. For comparison purposes, numerical specimens of the HSPs have identical mass (*T*_*f*_ = 1.0 mm, *T*_*c*_ = 50 mm, and $\overset{-}{\rho }=0.15$) and unit cell wall lengths (*h* = 8 mm and *l* = 4 mm), but different unit cell angles (−15°, −30°, −45°, −60°, and −75°) and corresponding wall thicknesses (0.336 mm, 0.260 mm, 0.183 mm, 0.113 mm, and 0.054 mm).  Figure 18(a) shows the projectile residual velocities after perforating the auxetic HSPs with reentrant unit cells featuring different cell angles. Clearly, for the same impact velocity, the residual velocities are varied for various cell angles. Different from the monotonic effects of macroscopic parameters (face and core thicknesses and core relative density) on the panels' responses (refer to Figures 14–17), variation of the mesoscopic parameter such as the reentrant unit cell angle leads to nonmonotonic response of the sandwich structure to ballistic impact. In the present case, a unit cell angle of *λ* = −60° yields the lowest residual velocity of the projectile at all impact velocities investigated. Moreover, the effect of cell angle on ballistic resistance is more profound for low velocity impact than for high velocity impact. A quartic polynomial approximation function was constructed to predict the projectile residual velocity within the examined ranges of cell angle and impact velocity, as plotted in Figure 18(b). The approximation surface also indicates the nonmonotonic influence of cell angle on the residual velocity, which achieves the lowest value along the black dotted line in the plot representing unit cell configurations with cell angle around −60°. Using (3), ballistic limits of the auxetic HSPs possessing various angled reentrant unit cells were achieved and plotted in Figure 18(c). No surprise, cell angle of −60° gives the highest ballistic limit value of *V*_*b*_ = 243.63 m/s, which is 35.97% higher than that of the panel with cell angle of −15°, which is 179.18 m/s. The ballistic limits and minimum perforation energy of the five auxetic HSPs with different reentrant unit cell angles and wall thicknesses are summarized in Table 4. It is worth noting that the minimum perforation energy for specimen No. 4 (*λ* = −60°) (*E*_*p*_ = 42.53 J) is almost twice that of specimen No. 1 (*λ* = −15°) (*E*_*p*_ = 23.0 J), implying again the great influence of reentrant unit cell angle on the ballistic capability of the auxetic HSP (Figure 18(d)). 

**Figure 19:**
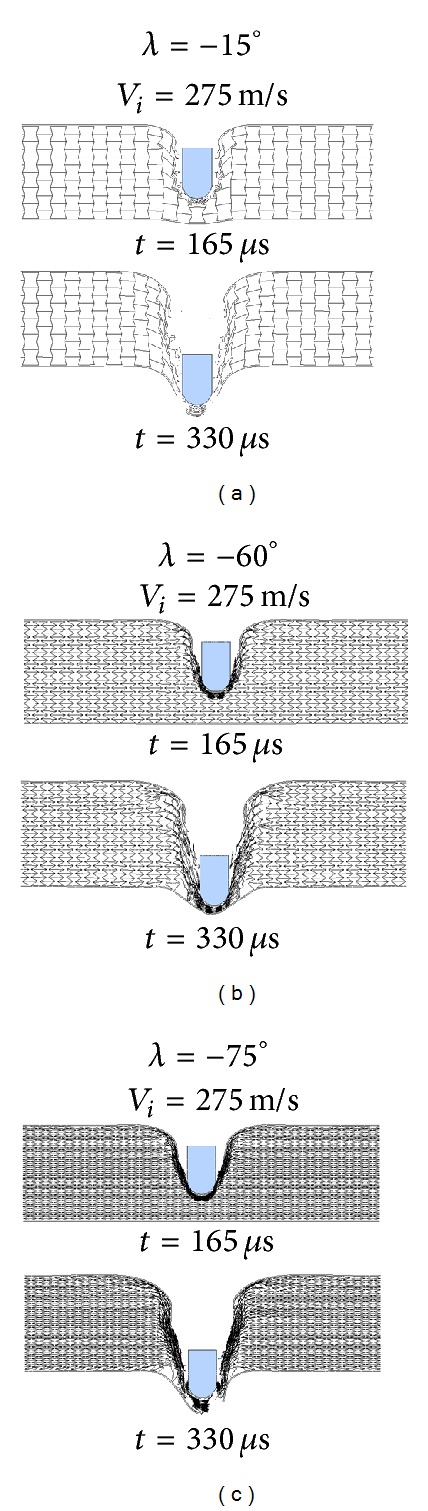
Snapshots of impact processes of auxetic HPSs with different reentrant unit cell angles by projectiles at *V*_*i*_ = 275 m/s: (a) *λ* = −15°, (b) *λ* = −60°, and (c) *λ* = −75°.

**Figure 20:**
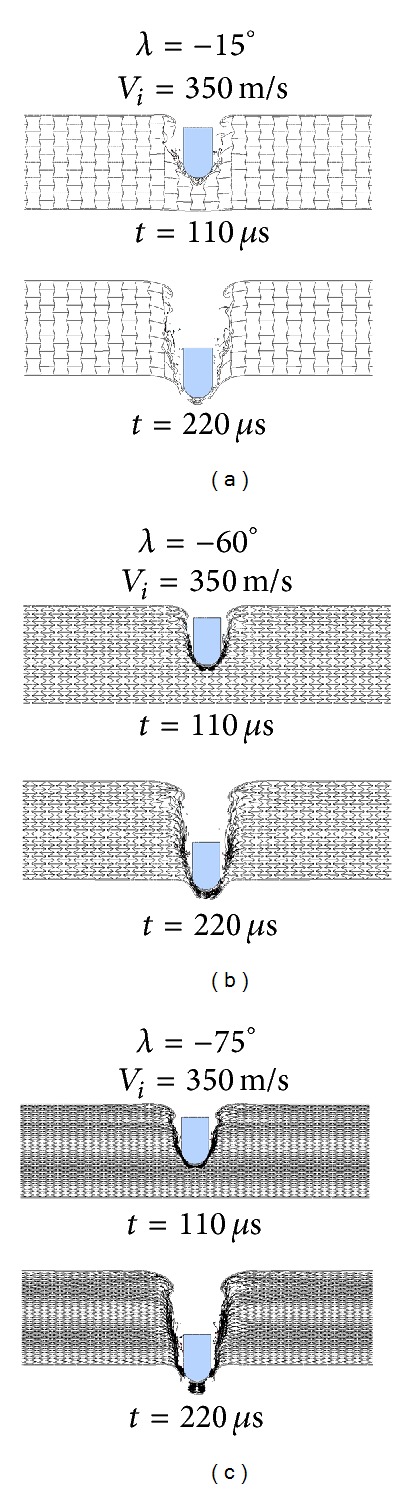
Snapshots of perforation processes of auxetic HPSs with different reentrant unit cell angles by projectiles at *V*_*i*_ = 350 m/s: (a) *λ* = −15°, (b) *λ* = −60°, and (c) *λ* = −75°.

An explanation for the nonmonotonic ballistic response of the auxetic HSP to unit cell shape change is proposed. With a small cell angle, for example, *λ* = −15°, the A-type HSP is more close to an R-type HSP (Figure 19(a)), while the latter is more prone to cell wall buckling and shearing (refer to Figures 7(a) and 8(a)) and has the least ballistic resistance among the three HSP configurations, as discussed in previous section. Enlarging the cell angle (absolute value) makes it easier for the inclined walls of the reentrant unit to rotate around the connecting edges of the cell walls, and thus bring the auxetic effect of the honeycomb core into play thoroughly. Large cell angle also helps to reduce the unit's vertical dimension and increase the number of units along the thickness direction of the honeycomb core. The foregoing two aspects, give a contribution for the high ballistic capacity of the auxetic HSP with −60° core unit cell angle (Figure 19(b)). On another hand, too large cell angle results in much thinner cell wall with reduced strength, which can be easily stretched to failure (Figure 19(c)). Therefore, a cell angle of  *λ* = −75° incurs even lower ballistic limit than a cell angle of *λ* = −45° (Figure 18(c)). As impact velocity increases, the ballistic damage to the HSP tends to be more localized (Figure 20), and the unit cell angle shows less effect (Figure 18(a)). 

#### 5.2.2. Effect of Unit Cell Size and Wall Thickness

**Figure 21:**
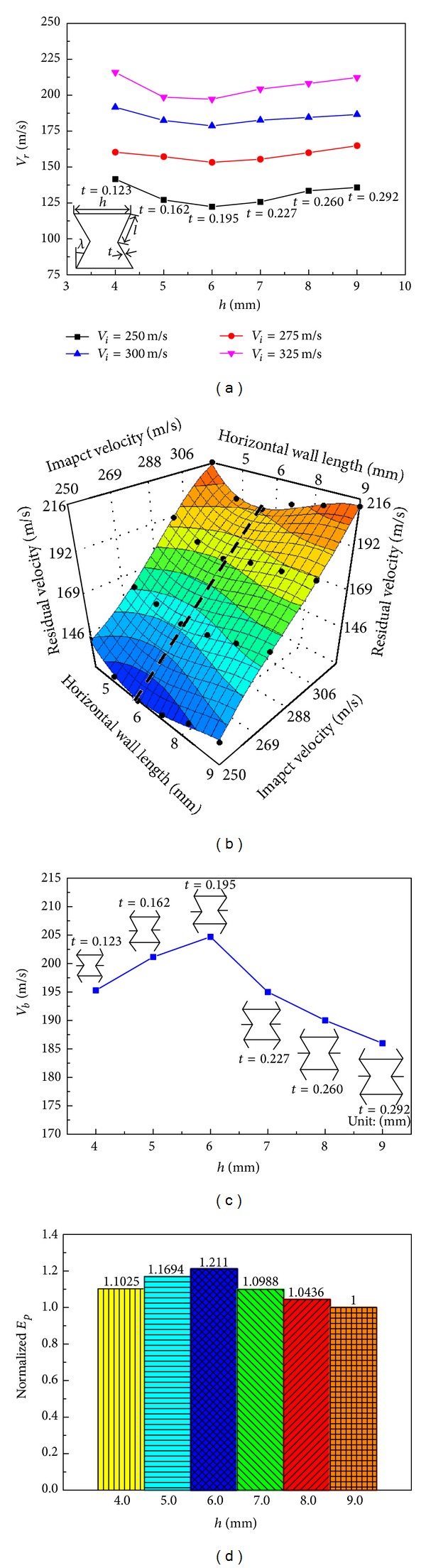
Effects of reentrant unit cell size and wall thickness on the ballistic responses of auxetic HSPs: (a) residual velocity variations with varied cell size and wall thickness at different impact velocities; (b) quartic polynomial approximation surface of residual velocity (The bold black dots in the plot show the training points); (c) ballistic limit versus unit cell horizontal wall length; and (d) normalized minimum perforation energy versus unit cell horizontal wall length. (All numerical specimens have *T*_*f*_ = 1.0 mm, *T*_*c*_ = 50 mm, $\overset{-}{\rho }=0.15$, and *λ* = −30°).

Now consider the effect of cell size of the reentrant unit of the honeycomb core, or the relative dimension of the unit cell and the projectile. With the same value of mass as that in Section 5.2.1, all simulated cases involved HSPs with *T*_*f*_ = 1.0 mm, *T*_*c*_ = 50 mm, $\overset{-}{\rho }=0.15$ and *λ* = −30°. The ballistic capabilities were numerically evaluated for six auxetic HSPs with the horizontal wall length *h* varying from 4.0 mm to 9.0 mm at a step size of 1.0 mm, the inclined wall length *l* half of the horizontal wall length, that is, *l* = *h*/2, and the corresponding wall thicknesses as listed in Table 5.  Figure 21 graphically shows the projectile residual velocities, ballistic limits and minimum perforation energy of the auxetic HSPs with different honeycomb core unit cell sizes. It is interesting to note that like the cell angle, unit cell size also shows nonmonotonic effects on the panels' ballistic responses, though the effects are relatively insignificant. Under the current circumstance, a unit cell with horizontal wall length *h* = 6.0 mm results in the best ballistic performance of the auxetic HSP. 

**Figure 22:**
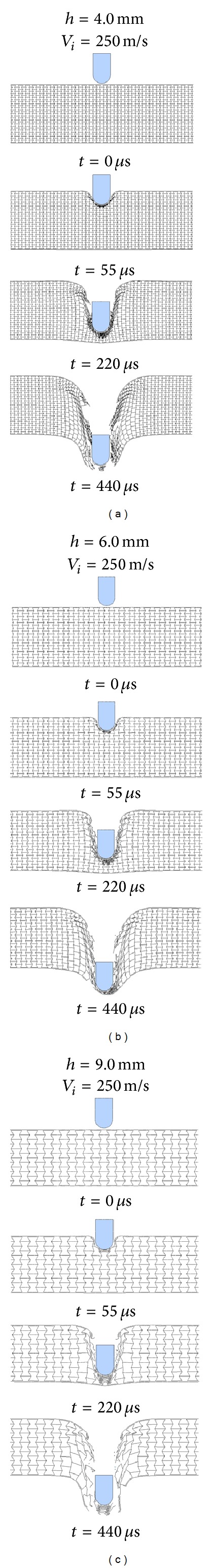
Perforation processes of auxetic HPSs with different reentrant unit cell sizes by projectiles at *V*_*i*_ = 250 m/s: (a) *h* = 4.0 mm, (b) *h* = 6.0 mm, and (c) *h* = 9.0 mm.

Snapshots of the perforation processes of auxetic HSPs with different honeycomb core unit cell sizes are shown in Figure 22. For a certain value of the honeycomb core relative density, a small unit cell corresponds to a thinner wall. On the one hand, downsizing the unit cell (or reducing the relative dimension of the unit cell and the projectile) makes more units be affected by the projectile, thereby manifesting the auxetic characteristics of the honeycomb core for enhanced ballistic capability (Figure 22(a)). On the other hand, as above-mentioned, thinner cells are easier to be stretched to failure.  Figure 22(b) reveals that a good balance is achieved between unit cell size (i.e. material concentration) and wall thickness (i.e. material strength) by a unit cell with horizontal wall length of *h* = 6 mm in response to a hemispherical nose projectile with diameter *D*_*p*_ = 15 mm.

Based on the results of Section 5.2.1 and this section, we may propose an optimal design of the reentrant unit cell of the honeycomb core with cell angle *λ* = −60° and horizontal wall length *h* = 6 mm to maximize the ballistic limit of an auxetic HSP for a given mass subjected to projectile impact as specified. It needs to be noted that this “optimal design” is limited to a relatively narrow scope, and a “global” one still needs to be sought through design optimization considering wider design domains and the interactions between different design parameters. 

### 5.3. Effect of Projectile Nose Shape on Auxetic HSP Ballistic Response

Effect of projectile profile is an important subject in ballistic mechanics, and several studies have been performed for sandwich structures with polymeric [34–36] and metallic [37] foam cores. As such, a numerical study is finally carried out to explore the effect of projectile nose shape on the impact behavior of the auxetic HSPs. Projectiles with three different nose shapes (conical, hemispherical, and blunt) were considered in the simulations with a similar mass.

**Figure 23:**
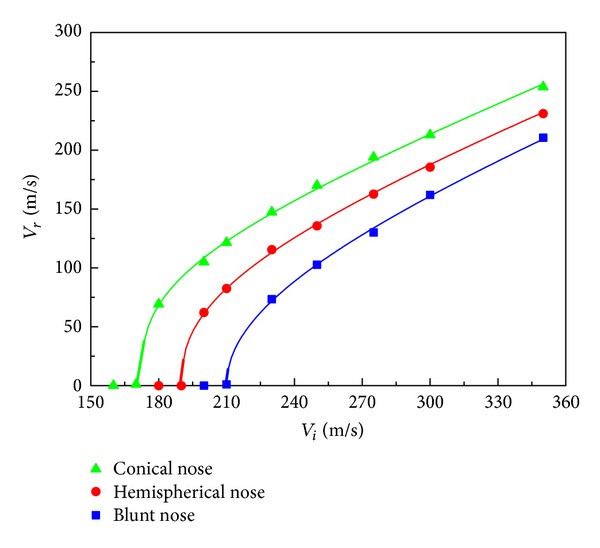
Residual velocity versus impact velocity of projectiles for auxetic HSPs with three types of nose shapes.

**Figure 24:**
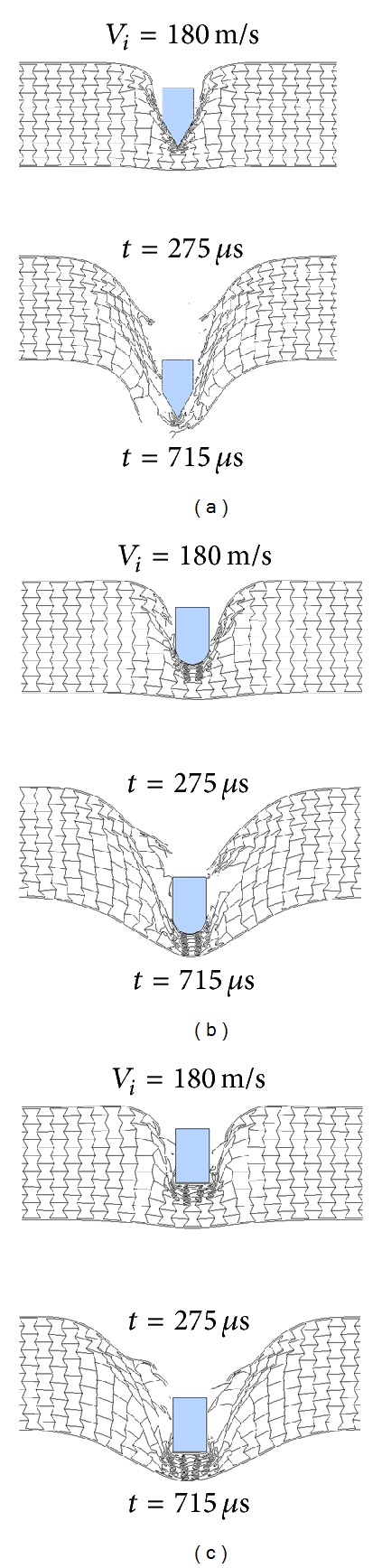
Impact processes of auxetic HSPs by (a) conical, (b) hemispherical, and (c) blunt nose projectiles at *V*_*i*_ = 180 m/s.

Residual velocity curves from numerical simulations of the three different projectiles impacting on the A-type HSPs with a baseline configuration (*T*_*f*_ = 1.0 mm, *T*_*c*_ = 50 mm, *ρ* = 0.15, *λ* = −30°, *h* = 8.0 mm, and *l* = 4.0 mm) are plotted in Figure 23. The figure reveals that blunter projectiles lead to lower residual velocities and higher ballistic limits. Ballistic limits of the auxetic HSPs subjected to conical, hemispherical, and blunt nose projectiles are 172 m/s, 190 m/s, and 210 m/s, respectively. Furthermore, it is seen that the influence of projectile shape becomes smaller with increased impact velocity, as pointed out in [12]. The results can be possibly explained as follows: blunt nose projectile has a much larger contact area with the auxetic honeycomb core material below its tip, and this causes more adjacent material compressed inwards due to the negative Poisson's ratio effect (Figure 24(c)), while the conical tip tends to push the material sideways and counteract to some extent the indentation resistance enhancement of the auxetic material, see Figure 24(a). At high impact velocity, due to the evolution of stress waves in the honeycomb, the mode of deformation and fracture in the core are localized, and less affected by the projectile profile. Additional in-depth study of this phenomenon is complex and open for further investigation.

## 6. Conclusion

In this paper, the dynamic responses of honeycomb sandwich panels (HSPs) under in-plane ballistic impact have been investigated numerically. The sandwich panels consist of two identical aluminum alloy face-sheets and an aluminum alloy honeycomb core featuring three different unit cell configurations: regular (H-type), rectangular-shaped (R-type), and reentrant or auxetic (A-type) hexagons. Finite element (FE) models of these three types of HSPs subjected to high-velocity hemispherical projectiles were developed and the impact processes were simulated by using the nonlinear FE code LS-DYNA. Three stages of the impact process describing perforation of the front face-sheet, honeycomb core, and back face-sheet have been clearly identified for the HSPs. Different from the out-of-plane impact of sandwich panels when most of the impact energy is absorbed by the face-sheets [9, 15], it is found that the honeycomb core absorbs the vast majority (over 80%) of the projectile kinetic energy under in-plane impact scenarios. The numerical results also show that the cell configuration affects greatly the dynamic response of the honeycomb core under projectile impact. As a result, the auxetic (A-type) HSP has the best in-plane ballistic performance among the three types of HSPs for the complete range of impact velocity (150 m/s–350 m/s) under investigation, even though the superiority of the A-type HSP is much more evident at low velocity impact than at high velocity impact; in particular, with the same core relative density, the ballistic limit of the A-type HSP is 18.0% and 8.6% higher than that of the R-type and H-type HSPs, respectively. Detailed FE analyses of the penetration process of the HSPs reveal that the enhanced perforation resistance of the auxetic honeycomb core is attributed to the following two mechanisms: the adjacent material concentration below the projectile tip due to negative Poisson's ratio effect and the transformation of tension loads to remote units of the honeycomb core due to cell wall inclination. 

To obtain an insight into the ballistic responses of the auxetic HSPs and quantify the projectile residual velocities, ballistic limits and hence minimum perforation energy of the panels, parameter study have been carried out for variations in both macroscopic parameters (face-sheet and core thicknesses, core relative density) and mesoscopic parameters (unit cell angle and size) of the structure itself as well as the projectile profiles. The main findings and design information from the study can be outlined as follows.

1. The residual velocity of the projectile decreases monotonically as the face-sheet thickness increases for a given core thickness and relative density. However, this decrease in the residual velocity is less significant for HSPs with thicker honeycomb cores compared with panels with thinner cores.
2. The residual velocity decreases with increased honeycomb core relative density for given core and face-sheets thicknesses. With increased core thickness, the residual velocity shows more nonlinearity in response to core relative density and the impact velocity.
3. For the considered honeycomb core thicknesses, the ballistic limit of the auxetic HSP is almost linearly proportional to both face-sheet thickness and core relative density, while the minimum perforation energy has nearly quadratic relationships with the two variables. Increasing the face thickness is more effective for ballistic limit (perforation energy) increment of the auxetic HSP with a thinner core. However, increasing the core relative density is more effective in improving the ballistic limits (perforation energy) of the thicker cored sandwich panels.
4. Variations of the mesoscopic parameters including the reentrant unit cell angle and cell size leads to nonmonotonic ballistic responses of the auxetic HSPs. In the present study, a reentrant unit cell angle of −60° and a horizontal wall length of 6 mm give the highest ballistic limits and minimum perforation energy due to the trade-offs between cell concentration and cell wall material strength. Compared with cell size, cell angle has relatively larger influences, especially at impact velocities near the ballistic limits.
5. Quartic polynomial functions can properly approximate projectile residual velocities in response to different impact velocities and various parameters, and may be used for response predictions.
6. Projectile nose shape affects greatly the ballistic curve of the auxetic HSPs; blunter nose results in higher ballistic limit of the auxetic HSP.

In the light of numerical simulations, this study shows promising results of using honeycombs with auxetic characteristics as sandwich core material for in-plane ballistic protection. Additional study such as optimization design of such structures with the intent to further increase their performances is suggested.

## Figures and Tables

**Table 1:** Values of empirical parameters in (3) and ballistic limits for the three types of HSPs.

Core configuration	*A*	*p*	Ballistic limit *V*_*b*_ estimated by (3) (m/s)
R-type hexagon	0.692	3.338	161
H-type hexagon	0.697	3.189	175
A-type hexagon	0.726	2.492	190

**Table 2:** Specifications and numerical results of three groups of auxetic HSPs, each of which has identical core thickness (*T*_*c*_ = 30 mm, 50 mm and 70 mm, respectively) and relative density ($\overset{-}{\rho }=0.15$), but different face thicknesses (*T*_*f*_ = 0.5 mm, 0.75 mm, 1.0 mm, 1.25 mm and 1.5 mm, respectively), so that the effect of core and face thickness can be studied.

Group no.	Face-sheet thickness, *T*_*f*_ (mm)	Core thickness, *T*_*c*_ (mm)	Core relative density, $\overset{-}{\rho }$	Ballistic limit, *V*_*b*_ (m/s)	Minimum perforation energy, *E*_*p*_ (J)
1	0.5	30	0.15	127.78	11.70
1	0.75	30	0.15	137.29	13.50
1	1.0	30	0.15	150.00	16.12
1	1.25	30	0.15	168.58	20.36
1	1.5	30	0.15	174.60	21.84
2	0.5	50	0.15	163.98	19.27
2	0.75	50	0.15	175.00	21.94
2	1.0	50	0.15	190.02	25.87
2	1.25	50	0.15	195.00	27.24
2	1.5	50	0.15	211.45	32.04
3	0.5	70	0.15	219.54	34.53
3	0.75	70	0.15	230.13	37.95
3	1.0	70	0.15	237.67	40.47
3	1.25	70	0.15	239.75	41.18
3	1.5	70	0.15	259.32	48.18

**Table 3:** Specifications and numerical results of three groups of auxetic HSPs, each of which has identical core thickness (*T*_*c*_ = 30 mm, 50 mm and 70 mm, respectively) and face-sheet thickness (*T*_*f*_ = 1.0 mm), but different core relative densities ($\overset{-}{\rho }$ = 0.1, 0.15, 0.18 and 0.2, respectively), so that the effect of core thickness and relative density can be studied.

Group no.	Face-sheet thickness, *T*_*f*_ (mm)	Core thickness, *T*_*c*_ (mm)	Core relative density, $\overset{-}{\rho }$	Ballistic limit, *V*_*b*_ (m/s)	Minimum perforation energy, *E*_*p*_ (J)
1	1.0	30	0.1	147.29	15.54
1	1.0	30	0.15	150.00	16.12
1	1.0	30	0.18	158.41	17.98
1	1.0	30	0.2	164.09	19.29
2	1.0	50	0.1	176.28	22.26
2	1.0	50	0.15	190.02	25.87
2	1.0	50	0.18	200.08	28.68
2	1.0	50	0.2	208.26	31.08
3	1.0	70	0.1	212.22	32.27
3	1.0	70	0.15	237.67	40.47
3	1.0	70	0.18	256.76	47.24
3	1.0	70	0.2	274.04	53.81

**Table 4:** Specifications and numerical results of a group of auxetic HSPs with identical mass (*T*_*f*_ = 1.0 mm, *T*_*c*_ = 50 mm, and $\overset{-}{\rho }$ = 0.15) and unit cell wall lengths (*h* = 8 mm and *l* = 4 mm), but different cell angles and wall thicknesses, so that the effects of cell angle and wall thickness can be studied.

No.	Cell angle, *λ* (deg)	Cell wall thickness, *t* (mm)	Ballistic limit, *V*_*b*_ (m/s)	Minimum perforation energy, *E*_*p*_ (J)
1	−15	0.336	179.18	23.00
2	−30	0.260	190.02	25.87
3	−45	0.183	222.99	35.63
4	−60	0.113	243.63	42.53
5	−75	0.054	211.40	32.02

**Table 5:** Specifications and numerical results of a group of sandwich panels with identical mass (*T*_*f*_ = 1.0 mm, *T*_*c*_ = 50 mm and $\overset{-}{\rho }$ = 0.15) and unit cell angle (*λ* = −30°), but different cell wall lengths and thicknesses, so that the effects of cell size and wall thickness can be studied.

No.	Horizontal cell wall length, *h* (mm)	Inclined cell wall length, *l* (mm)	Cell wall thickness, *t* (mm)	Ballistic limit, *V*_*b*_ (m/s)	Minimum perforation energy, *E*_*p*_ (J)
1	4.0	2.0	0.123	195.29	27.33
2	5.0	2.5	0.162	201.15	28.99
3	6.0	3.0	0.195	204.69	30.02
4	7.0	3.5	0.227	195.00	27.24
5	8.0	4.0	0.260	190.02	25.87
6	9.0	4.5	0.292	185.99	24.79

## Appendix

The quartic polynomial functions of projectile residual velocity *V*_*r*_ with respect to impact velocity *V*_*i*_ and face-sheet thickness *T*_*f*_ in this study are provided below.

For *T*_*c*_ = 30 mm, $\overset{-}{\rho }=0.15$ (Figure 14(a)):

(A.1) Vr(Vi,Tf)=3863.66−2445.07Tf−56.9542Vi+1951.61Tf2+13.9426TfVi+0.3442Vi2−1509.15Tf3+2.16319Tf2Vi−0.06067TfVi2−8.9799e−4Vi3+315.1122Tf4+0.8429Tf3Vi−9.1545e−3Tf2Vi2+9.7852e−5TfVi3+8.589e−7Vi4.

For *T*_*c*_ = 50 mm, $\overset{-}{\rho }=0.15$ (Figure 14(b)):

(A.2) Vr(Vi,Tf)=1.3814e4+60.6742Tf+218.6239Vi+673.7187Tf2−7.2208TfVi−1.2762Vi2−766.2974Tf3+3.7502Tf2Vi+0.01773TfVi2+3.3225e−3Vi3+153.2505Tf4+0.4279Tf3Vi−9.67e−3Tf2Vi2−2.69e−6TfVi3−3.25e−6Vi4.

For *T*_*c*_ = 70 mm, $\overset{-}{\rho }=0.15$ (Figure 14(c)):

(A.3) Vr(Vi,Tf)=2.0219e4+1921.056Tf−329.2659Vi+1962.78Tf2−36.4636TfVi+2.0017Vi2−383.5048Tf3−8.749Tf2Vi+0.1606TfVi2−5.3321e−3Vi3+26.1282Tf4+0.4561Tf3Vi+0.01362Tf2Vi2−2.1922e−4TfVi3+5.2667e−6Vi4.

The quartic polynomial functions of projectile residual velocity *V*_*r*_ with respect to impact velocity *V*_*i*_ and core relative density $\overset{-}{\rho }$ in this study are provided below.

For *T*_*c*_ = 30 mm, *T*_*f*_ = 1.0 mm (Figure 16(a)):

(A.4) Vr(Vi,ρ−)=1371.5+8372.629ρ−−28.8286Vi−5.8756e4ρ−2−29.3579ρ−Vi+0.1972Vi2+8.6074e4ρ−3+279.0165ρ−2Vi−0.04657ρ−Vi2−5.34e−4Vi3−3.299e4ρ−4+514.3329ρ−3Vi−0.9771ρ−2Vi2+4.1887e−4ρ−Vi3+4.868e−7Vi4.

For *T*_*c*_ = 50 mm, *T*_*f*_ = 1.0 mm (Figure 16(b)):

(A.5) Vr(Vi,ρ−)=−1.7159e4+1.1905e4ρ−+270.97Vi+2.8073e4ρ−2−193.253ρ−Vi−1.5719Vi2−1.4009e6ρ−3+2158.6347ρ−2Vi−0.3992ρ−Vi2+4.2526e−3Vi3+4.151e6ρ−4−4406.36ρ−3Vi−0.2077ρ−2Vi2+4.6745e−4ρ−Vi3−4.2691e−6Vi4.

For *T*_*c*_ = 70 mm, *T*_*f*_ = 1.0 mm (Figure 16(c)):

(A.6) Vr(Vi,ρ−)=−4.2326e4+1.8963e5ρ−+566.094Vi−1.1228e6ρ−2−860.7986ρ−Vi−3.142Vi2+4.645e6ρ−3+407.03ρ−2Vi+2.8585ρ−Vi2+7.8446e−3Vi3−7.2258e6ρ−4−238.1948ρ−3Vi−0.7159ρ−2Vi2−3.0263e−3ρ−Vi3−7.4341e−6Vi4.

The quartic polynomial functions of projectile residual velocity *V*_*r*_ with respect to impact velocity *V*_*i*_ and unit cell angle *λ* in this study are provided below.

For *T*_*f*_ = 1.0 mm,  *T*_*c*_ = 50 mm, $\overset{-}{\rho }=0.15$, *h* = 8 mm, and *l* = 4 mm (Figure 18(b)):

(A.7) Vr(Vi,λ)=3.4025e6+111.6234λ−4.392e4Vi−0.6398λ2−0.9285λVi+211.99Vi2−1.32e−3λ3+4.5107e−3λ2Vi+2.3978e−3λVi2−0.4535Vi3+3.5216e−5λ4−1.335e−5λ3Vi−4.7603e−6λ2Vi2−2.0825e−6λVi3+3.6288e−4Vi4.

The quartic polynomial functions of projectile residual velocity *V*_*r*_ with respect to impact velocity *V*_*i*_ and unit cell horizontal wall length *h* in this study are provided below.

For *T*_*f*_ = 1.0 mm,  *T*_*c*_ = 50 mm, $\overset{-}{\rho }=0.15$, *l* = *h*/2, and *λ* = −30° (Figure 21(b)):

(A.8) Vr(Vi,h)=1.059e5−1948.34h−1442.289Vi+42.5836h2+18.083hVi+7.3678Vi2+1.678h3−0.3579h2Vi−0.0548hVi2−0.0167Vi3−0.03442h4−4.829e−3h3Vi+7.8466e−4h2Vi2+5.1667e−5hVi3+1.4209e−5Vi4.
